# Distribution Patterns of Cocaine- and Amphetamine-Regulated Transcript- and/or Galanin-Containing Neurons and Nerve Fibers Located in the Human Stomach Wall Affected by Tumor

**DOI:** 10.3390/ijms19113357

**Published:** 2018-10-26

**Authors:** Anna Kozłowska, Janusz Godlewski, Mariusz Majewski

**Affiliations:** 1 Department of Human Physiology, School of Medicine, Collegium Medicum, University of Warmia and Mazury in Olsztyn, Warszawska Av 30, 10-082 Olsztyn, Poland; mariuszm@uwm.edu.pl; 2 Department of Human Histology and Embryology, School of Medicine, Collegium Medicum, University of Warmia and Mazury in Olsztyn, Warszawska Av 30, 10-082 Olsztyn, Poland; janusz350@poczta.onet.pl

**Keywords:** cocaine- and amphetamine-regulated transcript, galanin, cancer, stomach

## Abstract

The aim of the study was to investigate the distribution patterns of cocaine- and amphetamine-regulated transcript- (CART-) and galanin-immunoreactive (GAL-IR) neuronal structures in the human stomach wall, focusing on differences observed in regions directly affected by the cancer process, and those from the surgical margin. Samples from the stomach wall were collected from 10 patients (3 women and 7 men, the mean age 67.0 ± 11.9). Next, triple-immunofluorescence staining was used to visualize the changes in the frequency of neurons inside myenteric plexi and intramural fibers containing CART and/or GAL, as well as protein gene product 9.5 (as panneuronal marker). Tumor into the stomach wall caused a decrease in the number of CART-positive (+) nerve fibers in the longitudinal (LML) and circular muscle layers (CML). Notable changes in the dense network of CART+/GAL+ nerve fibers (an increase) were observed in the LML and lamina muscularis mucosae (LMM) within carcinoma-affected areas of the human stomach. Additionally, an elevated number of these nerve fibers from LMM were accompanied by an increase in the number of fibers containing GAL in the vicinity of the neoplastic proliferation. Obtained results suggest that a carcinoma invasion may affect the innervation pattern of the human stomach wall and their function(s).

## 1. Introduction

The digestive system is controlled and coordinated by the enteric nervous system (ENS) together with the central nervous system (CNS). The connections between the ENS and CNS are carried by the vagus and pelvic nerves, and sympathetic pathways. It was reported that role of the ENS and CNS differ considerably along the digestive tract. In the case of the ENS, it was shown that, in the small intestine and colon, it controlled, e.g., muscle activity, transmucosal fluid fluxes, and local blood flow [1,2]. The stomach ENS is mainly engaged in the regulation of peristaltic waves which are responsible for grinding and emptying [3]. On the other hand, CNS has a major role in monitoring the state of the stomach and, in turn, controlling its contractile activity and acid secretion, through vagovagal reflexes, as well as control of defecation [1,2].

The ENS of the human gastrointestinal tracts is strongly represented by intramural nerve fibers and neurons which are distributed in numerous ganglia. Most of these nerve cell bodies are situated in the myenteric plexi (MP; Auerbach’s plexi), and they form a network that extends from the upper esophagus to the internal anal sphincter. Whereas submucosal ganglia and connecting fiber bundles form submucosal plexi (Meissner’s plexi) in the small and large intestines, but not in the stomach and esophagus [1]. In the digestive system, the neurons from MP supply mainly smooth muscle control their activity, whereas those located in the SP regulate mucosal secretion and blood flow [1,4,5,6,7,8]. On the other hand, in the stomach, there also exists a large population of enteroendocrine cells producing peptidyl hormones, e.g., gastrin, secretin, pancreatic polypeptide, somatostatin, neurotensin, and ghrelin/motilin. These hormones play an important role in the regulation (stimulation or inhibition) of gastric acid secretion, motility, mucosal proliferation, gastric emptying, as well as visceral blood flow [9].

Recent studies showed that morphology and chemical coding of neurons, as well as nerve fibers of the human ENS in the stomach and intestine, can undergo changes under various pathological conditions. Namely, it was reported that the size of MP located in the stomach and intestinal wall in the vicinity of the tumor infiltration were significantly smaller compared to distally located plexuses [10,11,12]. For colorectal cancer, the atrophy of MP was accompanied by changes in the relative frequency of neuronally expressed proteins [13,14,15,16]. One of them is cocaine- and amphetamine-regulated transcript peptide (CART), which was identified in the gastrointestinal tract for the first time by Couceyro et al. [17]. Additionally, it was noted that the neoplastic infiltration of the intestine wall caused changes in the distribution of this peptide inside neuronal structures. It was reported that the relative frequency of CART-IR neurons in the ENS plexuses was increased in the human colon challenged by adenocarcinoma invasion. However, the density of nerve fibers containing CART within particular layers of the intestinal wall did not differ between macroscopically unchanged and cancer-affected region [16]. Moreover, it is well known that CART-IR neuronal structures in the ENS (plexus as well as nerve fibers) of the human and porcine stomach, during physiological state, simultaneously contain other neurotransmitters and/or neuromodulators, such as substance P, calcitonin gene-related peptide, galanin (GAL), vesicular acetylcholine transporter, leu-enkephalin, vasoactive intestinal peptide, neuropeptide Y, and nitric oxide synthase [5,18,19,20]. GAL, next to CART, seems to be the most important peptide during neoplastic process in the gastrointestinal tract. Recent studies have demonstrated that the relative frequency of GAL-IR neurons inside MP increase in the human colon and stomach wall affected by cancer [11,15]. Moreover, an increase in GAL expression, similar to CART, may be associated with poor prognosis of CRC [21,22].

Therefore, considering the findings described above, as well as a lack of data regarding the distribution of CART with GAL in ENS of the cancer-affected human stomach, this study aimed to investigate the presence of these peptides inside (a) MP and the muscle layers of a cancer-affected stomach wall, and (b) control tissue derived from the operative margin.

## 2. Results

### 2.1. Co-Localization of CART and GAL in Neurons of Myenteric Plexi (MP) in the Control and Cancer-Affected Areas of the Human Stomach Wall

**Figure 1:**
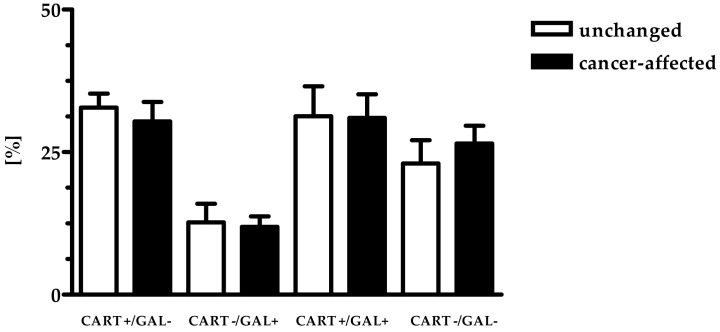
Relative frequency of particular subclasses of neurons presented as percentage (mean ± SD).

**Figure 2:**
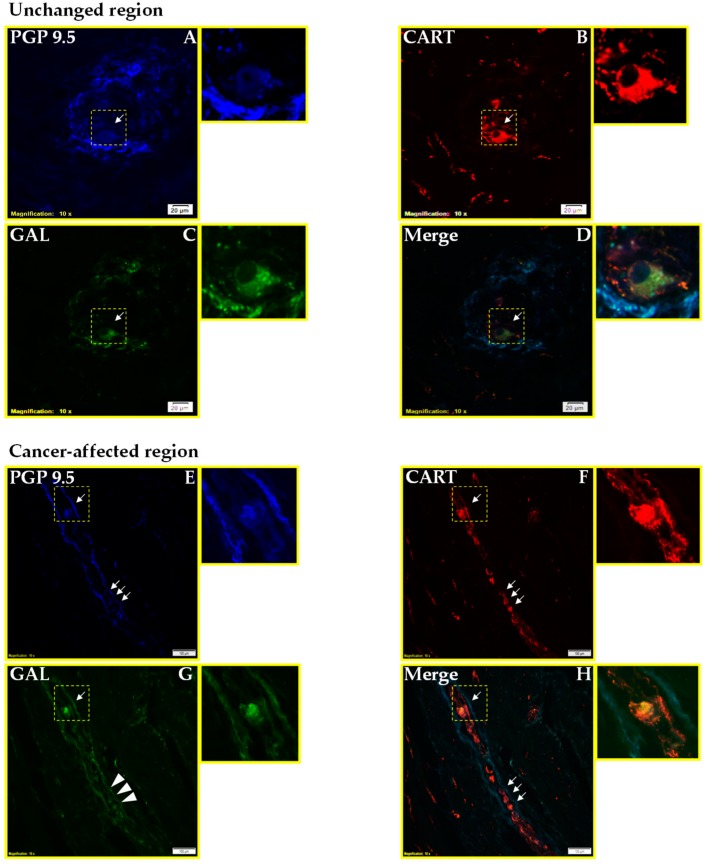
Micrographs showing myenteric plexi which were triple immunostained for cocaine- and amphetamine-regulated transcript (CART), galanin (GAL), and protein-gene product 9.5 (PGP 9.5; as a pan-neuronal marker). Small-sized arrows show double- or triple-stained cells, while large-sized arrows pointed out the lack of occurrence of studied substances. Micrographs D and H showing the superposition of all three channels: blue—PGP 9.5, red—CART, and green—GAL. In the macroscopically unchanged human stomach wall, one PGP 9.5-immunoreactive (-IR, (**A**)) neuron which was simultaneously CART-IR (**B**) and GAL-IR (**C**). In the cancer-affected human stomach wall, a moderate number of PGP 9.5-IR (**E**) neurons which were simultaneously CART-IR (**F**) but negative for GAL (**G**), and only one PGP 9.5-IR neuron which was simultaneously CART-IR (**F**) and GAL-IR (**G**). Colocalization of all antigens in the studied neuron (**D**,**H**). Scale bar = 20 µm and 100 µm.

Immunofluorescent staining showed that the percentages of PGP9.5+ neurons expressing CART and/or GAL were similar in both studied regions: close to tumor invasion and in the region distal from it (Table 1, Figure 1 and Figure 2).

### 2.2. Density of CART- and GAL-Expressing Fibers in the Muscular Layers in the Control and Cancer-Affected Human Stomach Wall

**Figure 3:**
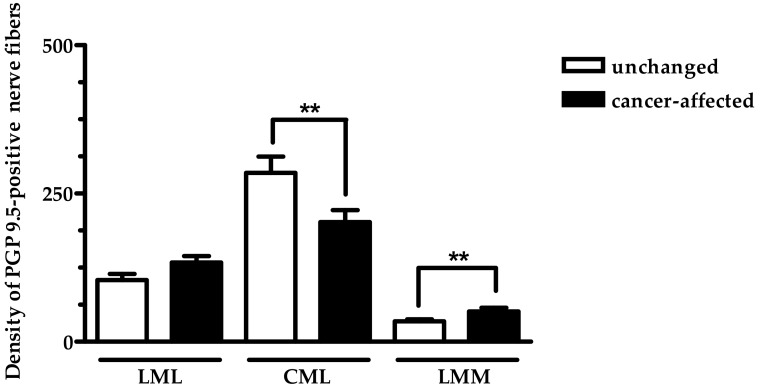
The mean number of PGP 9.5-positive nerve fibers counted in particular muscle layers of the surgical margin (open bars) and cancer-affected wall of the human stomach. Data were pooled and presented as the mean (range) of 10 patient. Abbreviations: LML—longitudinal muscle layer; CML—circular muscle layer; LMM—lamina muscularis mucosae. Data are presented as average number of nerve fibers (±SD) per area studied (900 µm × 700 µm). ** *p* < 0.01.

Next, we have elucidated the density of PGP 9.5-reactive nerve fibers in the particular muscle layers of control and cancer-affected stomach regions (Figure 3). Although in control tissue, PGP 9.5-positive nerve fibers were observed in all studied muscle layers, it should be stressed that they were the most numerous in the circular muscle layer (CML), when compared to their numbers in both the longitudinal muscle layer (LML) and lamina muscularis mucosae (LMM). Interestingly, as shown in Figure 3, while the number of PGP 9.5-positive nerve fibers observed in the LMM of the cancer-affected stomach wall region was higher than that observed in the LMM of the control specimens, PGP 9.5-positive nerve fibers supplying the CML were less numerous in cancer-affected regions than in control tissue. The average number of nerve fibers containing PGP 9.5 in the stomach LML did not significantly differ between surgical margin and cancer-affected regions of the organ.

**Figure 4:**
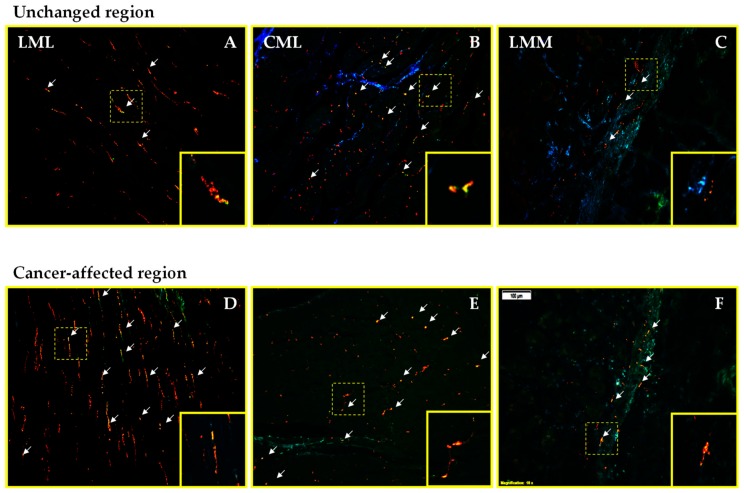
Micrographs showing nerve fibers triple immunostained for cocaine- and amphetamine-regulated transcript (CART), galanin (GAL), and protein-gene product 9.5 (PGP 9.5; as a pan-neuronal marker). Micrographs (**A**–**F**) showing the superposition of all three channels simultaneously: red—CART, green—GAL, and blue—PGP 9.5. Distribution pattern of nerve fibers immunoreactive for studied substances within the longitudinal (LML), circular (CML), and lamina muscularis mucosae (LMM) in the human stomach wall affected by cancer (Figure 4D–F, respectively) and control tissue derived from the operative margin (Figure 4A–C, respectively). Scale bar = 100 µm.

**Figure 5:**
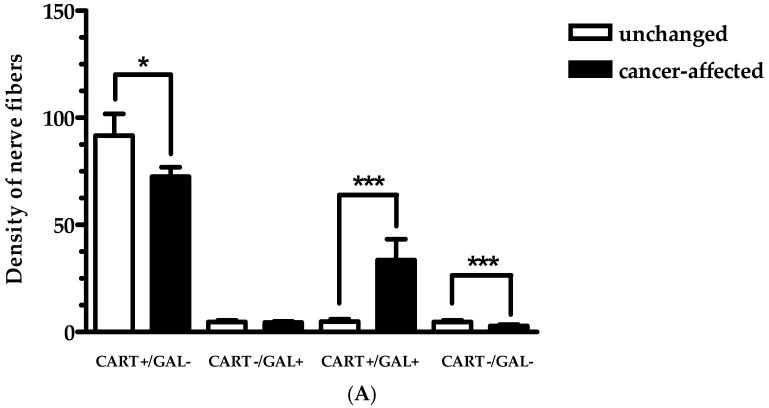

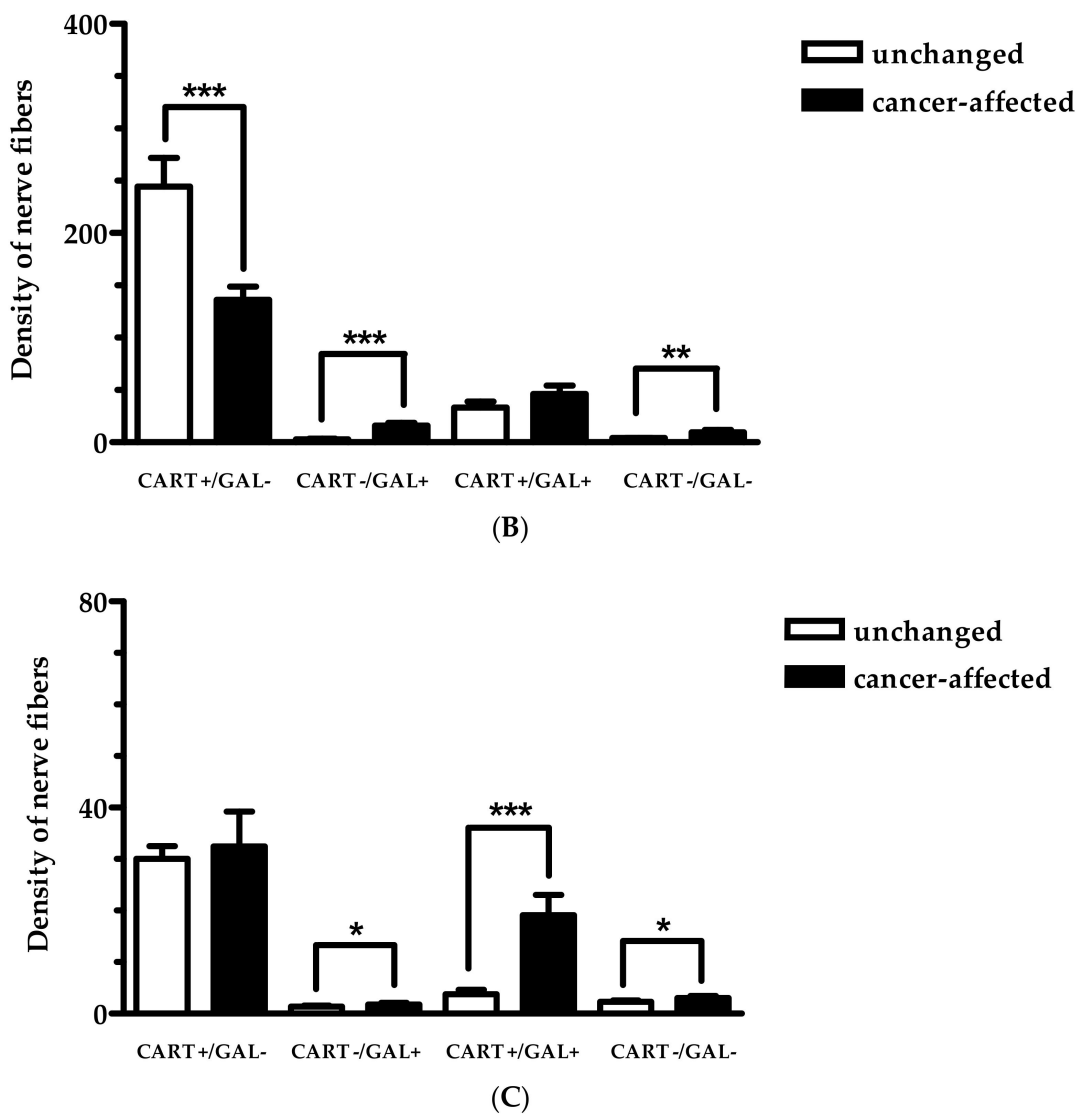
The mean density of nerve fibers containing CART and/or GAL in the longitudinal muscle layer (LML; (**A**)), circular muscle layer (CML; (**B**)) and lamina muscularis mucosae (LMM; (**C**)) in both cancer-affected and control tissue from human stomach wall. Data were pooled and presented as the mean (range) of 10 patients. Data are presented as average number of nerve fibers (±SD) per area studied (900 µm × 700 µm); *, **, *** are *p* < 0.05, *p* < 0.01, and *p* < 0.001, respectively.

In the case of CART+ and/or GAL+ nerve fibers, they also were observed in all muscle layers of the control tissue. It should be underlined that the leading subpopulation there was represented by fibers containing CART. Additionally, in the LML and LMM, the density of CART+/GAL+ nerve fibers was significantly lower in the surgical margin, compared to cancer-affected region (Figure 4A,D; Figure 4C,F; respectively). Moreover, triple-immunofluorescence staining revealed that the number of GAL+ nerve fibers in the CML and LMM distal from cancer invasion was lower than cancer-affected region. Meanwhile, the number of nerve fibers immunoreactive for CART+ in the LML and CML in the samples from surgical margin was significantly higher when compared to the tissue from cancer-affected region. In turn, the density of CART+/GAL+ nerve fibers in the CML (Figure 4B—cancer-free; Figure 4E—cancer-affected), CART+ nerve fibers in the LMM and GAL+ nerve fibers in the LML, were similar in both studied samples (Figure 5A–C).

## 3. Discussion

This is the first study that provides a description of the chemical phenotypes of neurons inside MP, as well as nerve fibers in the muscle layer distal from and in the vicinity of cancer invasion. Moreover, the present results suggest that neoplastic process in the human stomach is a factor evoking visible changes in the density of intramural nerve fibers.

### 3.1. Distribution Pattern of CART with GAL

The results of the present study showed that the distribution pattern of CART-IR neurons within MP did not differ significantly between tissue in proximity to cancer invasion, compared to unchanged parts of the stomach. These results were similar to that previously observed in a patient with colorectal cancer (CRC) [16]. Moreover, our results showed that cancer did not change the frequency of neurons containing CART with GAL, or devoid of CART but positive for GAL. There is no detailed data describing the distribution pattern of CART+/GAL+ neurons in the MP in the stomach wall affected by cancer. In the case of GAL, it has only been reported that, in the group of patients with stomach cancer, the percentage of the neurons containing GAL was lower in the caspase-3-positive subpopulation of neurons, while in the subpopulation of caspase-8-positive cells, such neurons were more numerous in the MP located close to tumor invasion [11].

The present study also suggests that neoplastic changes were associated with an increased number of PGP 9.5 nerve fibers in LMM, while it decreased the number of these fibers in the CML. The reason for the changes in the number of nerve fibers in proximity to tumor invasion is not fully understood in the human cancer-affected stomach wall. However, it may be expected that cancer cells utilize the neurotrophic factors released by the nerve fibers to create a positive microenvironment for cell survival, and also for their proliferation or/and release of these substances to stimulate their growth [23].

In the present study, in the cancer-affected stomach wall, the density of nerve fibers in the muscle layers immunoreactive to CART and/or GAL was clearly changed in proximity to tumor invasion, when compared to the unchanged area. Our studies have shown that, in the cancer-affected wall, there were visible increases in the number of CART+/GAL+ nerve fibers in the LML and LMM, as well as GAL-IR in the CML and LMM. Moreover, in the present study, the number of CART-IR nerve fibers in the CML and LML, in the vicinity of cancer invasion, was significantly lower compared to control areas. It is difficult to explain this phenomenon because, in the available literature, there is a lack of detailed data concerning the co-localization of CART with GAL in nerve fibers. Until now, the co-localization of CART with GAL was described only in the healthy human caecum [24]. In reference to CART, it has only been found that, in the cancer-affected colon wall, the number of CART-IR nerve fibers in the LML and LMM was similar compared to corresponding “healthy” tissue. However, in the CML in the proximity of the neoplastic process, the density of these fibers was significantly higher than that observed in the control tissue [16]. An increase in the number of CART-IR in all muscle layers of porcine stomach wall after T-2 toxin administration was also observed [25]. This discrepancy, between the results in our study and those of Oponowicz et al. [16] and Makowska et al. [25], can probably be explained by differences in the arrangement of nerve fibers in the gastrointestinal tract [26] and/or species-specific differences [19]. Additionally, it should be underlined that the results of the present study indicate that although tumor invasion had no influence on the number of PGP 9.5+ nerve fibers in the LML, it had a large impact on their chemical coding pattern, e.g., increase in the number of CART+/GAL+ nerve fibers. Changes in chemical coding of these nerve fibers, in the cancer-affected stomach tissue, may play an important role in the adaptation of the nervous system under pathological conditions [27]. In the case of GAL, there is no detailed data describing the distribution pattern of these nerve fibers in the cancer-affected stomach wall. It has only been reported that, in patients with CRC, GAL concentrations were higher within the muscular layer of the colon wall located in the vicinity of cancer invasion, compared to the sections distally from it [12].

Interestingly, in the preset work, the percentage of CART+ and/or GAL+ neurons was similar in both studied samples, whereas the number of nerve fibers containing these peptides significantly increased in the muscle layers of the cancer-affected region. This is probably associated with the fact that the gastric wall is largely innervated by extrinsic nerve fibers (vagal origin) [1], and this kind of nerve fiber contributes to gastric tumorigenesis [28,29]. The role of these nerve fibers underlines, also, a recent study which showed that denervation might suppress tumor progression, which was earlier reported in three independent mice models of gastric cancer [28].

### 3.2. Functional Considerations

It is generally accepted that, in the central nervous system, CART is involved in the control of food intake, body weight regulation, stress, and also, reward and pain transmission. It was recently reported that this peptide exerts a neuroprotective effect during neurological diseases [30,31,32]. However, the function of CART within the human ENS of the stomach is not fully understood [5]. Previous studies demonstrated that incubation of muscle strips from the rat stomach, with an addition of CART, did not change the motor activity of those strips [33]. On the other hand, it is known that, in rodents, the central administration of CART regulates gastric emptying and, also, reduces gastric acid secretion through corticotropin-releasing factor [34,35,36]. Furthermore, it is suggested that CART plays a neuroprotective and adaptive role within the ENS during pathological conditions [37]. For example, it was reported that CART suppressed neurotoxicity and enhanced neuronal survival after ischemia (oxygen and glucose deprivation) via a mitochondrial mechanism [37,38,39]. It seems to be likely that this peptide plays a role as a co-transmitter or neuromodulator. The results obtained in the present study, which showed the co-occurrence CART with GAL, seem to partly confirm this hypothesis. It is generally known that galanin-like peptide is also, similarly to CART, widely expressed throughout the gastrointestinal tract [40], predominantly in the myenteric and submucosal plexi [41,42]. Moreover, according to numerous studies, GAL is involved in the control of food intake [43,44,45] and appetite [43,46]. Furthermore, inhibitory effects of GAL on gastric acid secretion was reported in rats [47,48], and is a convergent result to that observed under the influence of CART, as described above. Previous studies also showed that GAL can modulate gastric motility in rats, depending upon the receptor subtype activation [49]. Moreover, GAL can be implicated in the survival of ENS neurons under pathological conditions. This supposition was strongly supported by data showing that this peptide is able to inhibit gastric carcinogenesis in rats [50] and in the human gastric cell lines, which can be impaired by its hypermethylation [51].

It should be underlined that changes in the neuronal subpopulations associated with CART and/or GAL availability could also impact on tumor growth without the influence on its grading. It was reported that in rats with gastric carcinogenesis induced by *N*-methyl-*N*′-nitro-*N*-nitrosoguanidine, prolonged administration of GAL significantly decreased the incidence of gastric and colon cancers without influence on the histological types of cancers. Authors suggested that this effect may be linked with suppression of antral epithelial cells and colonic epithelial-cell proliferation. Therefore, the enhanced number of GAL-positive nerve fibers within CML and LMM observed in this study may be related to the neuroprotective role of this peptide (especially with CART). However, this hypothesis needs to be further verified in detail [52,53]. In the case of CART, it was shown that elevated expression of this peptide is associated with worse survival of patients (probably associated with higher tumor cell viability under the influence of this peptide). Moreover, the changes in the CART expression were not related to disease stage and tumor grade [21].

It should not be excluded, participation in the cancer process of other substances produced in the stomach. For example, it was reported that neuronally expressed proteins are peptides regulating food intake and/or stomach motility under physiological condition [54]. Moreover, ghrelin increases the serum level of growth hormone secretion and insulin-like growth factor-1 (IGF-1), and plays an important role in the therapy of the stomach and duodenum ulcers in rats. Whereas, in the case of tumors, this peptide can promote their occurrence, especially in the gastrointestinal tract [55,56,57,58]. Meanwhile, obestatin, similarly to grelin, exhibited some protective and therapeutic effects in the stomach ulcers and duodenum colitis. With reference to gastric cancer, the obestatin stimulates mitogenesis of gastric cancer cells [59,60,61,62]. Therefore, further studies utilizing more neuronal markers (especially sensory markers), have to be carried out, in order to elucidate the relevance of different subpopulation of neurons innervating the stomach wall [63,64]. 

Prostaglandins should not be omitted from the list of substances which need to be studied in the next order, because they regulate the secretion of bicarbonate and mucous, inhibit gastric acid secretion, maintaining epithelial cell restitution and mucosal blood flow and, in this way, they play a key role in gastric epithelial defense [65]. These substances seem to be especially interesting because our unpublished data suggests that prostaglandins might be involved in the cancerogenesis. e.g., our preliminary data in stomach cancer-affected wall showed that MPO-positive cells were more numerous in the stomach wall close to cancer infiltration, whereas the number of CD38-positive cells was similar in the tissue localized close and distally from cancer.

On the other hand, in the present study, single CART-IR gastric submucosal neurons close to cancer invasion, and in the macroscopically unchanged tissue, were present. This result is congruent with previous studies in a patient with CRC [16], as well as those performed on the porcine stomach wall [24]. However, there were some differences. It was previously reported that, in the rat [5] as well as pig stomach [18,20], such neurons were not present. This discrepancy may be attributed to the production of this peptide within the submucosal plexi in the human stomach wall under pathological conditions. However, this hypothesis needs to be further verified in detail.

## 4. Materials and Methods

### 4.1. Ethical Statement

The samples from the present study were collected based on a protocol approved by the Bioethics Commission (No. 18/2012, 29 November 2012) of University of Warmia and Mazury in Olsztyn, Poland, and written informed consent was obtained from all patients in the study.

### 4.2. Patient Recruitment and Specimen Collection

The present study was performed using post-operative material derived from 10 patients with diagnosed cancer of the stomach, which was harvested during surgery at the Department of Oncological Surgery of the Regional Oncological Centre in Olsztyn, Poland. The study group consisted of 3 women and 7 men, the mean age of the patients was 67.0 ± 11.9 years (range from 51 until 85 years). The post-operative pathomorphological analyses confirmed that patients included in this study formed a homogenous group with the same degree of adenocarcinoma invasion within the stomach wall, defined as T3 on the TNM scale by the American Joint Committee on Cancer (AJCC) staging. Moreover, none of the patients had a second serious illness or neo-adjuvant chemo- and/or radiotherapy.

Directly after surgical organ resection, small samples (approximately 1 cm^2^) were obtained in duplicate from the same gross-anatomical localization (body of the stomach) in each patient enrolled in the study. Fragments of the stomach wall were collected from the region of cancer invasion and from a macroscopically-unchanged region, at a distance of 5–8 cm from the cancer (surgical margin; if neoplastic cells were not observed during histopathological examination, the sample was used as a control tissue) were fixed by immersion in the 4% buffered paraformaldehyde (pH 7.4) for 120 min, washed twice in 0.1 M phosphate buffer (pH = 7.4, 4 °C) over three days, and then stored in 18% buffered sucrose solution containing 0.01% sodium azide (pH = 7.4) for 7 days at 4 °C, until freezing and sectioning.

### 4.3. Experimental Procedures

#### 4.3.1. Immunofluorescence Procedures

Ten-mm-thick cryostat (HM525 Zeiss, Berlin, Germany) sections of the stomach wall were processed for triple-immunofluorescence staining technique, as described previously by Kozłowska et al. [10], using antibodies listed in Table 2.

The sections of stomach wall were incubated overnight in the humid chamber, with primary antibodies raised in different host-species: PGP 9.5 (used in the present study as panneuronal marker), CART, and GAL. The immunological complex was visualized with species-specific secondary antibodies labelled with AMCA- or FITC-conjugated secondary antiserum, and biotinylated donkey anti-rabbit antibody. The latter antibody was finally visualized by the additional incubation of sections with streptavidin-CY3 complex for 1 h. After a final wash, all sections were cover-slipped with carbonate-buffered glycerol (pH 8.6).

The specificity of primary antisera was tested by standard controls, including pre-absorption test (10 μg of antigen per 1 mL of diluted antiserum) on the sections from the stomach wall. The primary antibody was also omitted from the incubation, and normal rabbit, guinea pig, or mouse serum was substituted for the primary antibody. No specific immunostaining was observed by the specimens after replacement and omission of the respective primary antiserum in control (negative) probes.

#### 4.3.2. Counting Neurons and Nerve Fibers

Triple-immunolabelled neurons in the MP, as well as nerve fibers in the muscular layer, were analyzed under an Olympus BX61 microscope (Olympus, Tokyo, Japan) equipped with the epi-fluorescence kit and appropriate filter sets for AMCA (V1 module, excitation range 330–385 nm and barrier filter at 420 nm), FITC (B1 module, excitation filter 450–480 nm), and CY3 (G1, excitation filter 510–550 nm). Micrographs were acquired using 10× and 20× objectives, and a PC equipped with a CCD camera operated by Cell Sens Dimension image analyzing software (Olympus, Warsaw, Poland). The submucosal neurons were not analyzed because their number was too low to obtain reliable data (CART-IR neurons were found sporadically).

The mean number (±SD) of neurons within MP was counted in twelve cryostat sections (10 µm-thick) obtained from the two parts of the stomach wall (*n* = 6 samples from region in the vicinity of cancer invasion; *n* = 6 samples from surgical margin). The distance between these sections was always larger than 150 μm to avoid double counting of the same neuron within MP in adjacent sections. The population of CART- and/or GAL-IR neurons was subdivided into four subpopulations: those simultaneously containing CART, GAL, and PGP 9.5; those containing CART and PGP 9.5; or GAL and PGP 9.5; as well as those devoid of CART and GAL but containing PGP 9.5. The number of neurons immunoreactive for PGP 9.5 in the MP, as well as in the nerve fibers, was considered as the total number of neurons (100%). It should be underlined that all patients showed similar changes in density/number of CART+ and/or GAL+ fibers in diseased tissue.

The distribution and relative frequency of labelled nerve fibers were counted in ten cryostat sections (*n* = 5 samples from region in the vicinity of cancer invasion, *n* = 5 samples from surgical margin; in 5 fields per section) using the Merz grid from Fiji software [66]. All counts were made on coded slides prepared by the first author at 10× magnification using 900 µm × 700 µm regions as the test frames. To avoid fluorescence fading, a test frame was digitally recorded before counting. Such digital frames were in the form of stacks, which consisted of three micrographs representing red, green, and blue immunofluorescence channels. Saved stacks were then evaluated by two independent experimenters, being blind to the parameters of the studied tissue. The results of these counts showed high interrated reliability (Pearson *R* = 0.84, *p* < 0.01).

#### 4.3.3. Statistical Analysis

To ensure the reliability of the results, statistical analysis of the results obtained from all patients was performed. The non-parametric Mann–Whitney *U*-test was performed to evaluate the differences in the frequency of CART and/or GAL-IR neurons, as well as the density of nerve fibers containing these substances between control and cancer-affected samples. The Tukey ANOVA was used to estimate the differences in the density of CART- and/or GAL-IR between the particular muscle layers of cancer-affected and the control part of the stomach wall. In all performed analyses, the results were considered to be statistically significant (*p* < 0.05).

## 5. Conclusions

This is the first report on the co-localization pattern of CART and GAL in the neural structures of the human stomach, especially in regions affected by cancer infiltration. As may be judged from the observed changes in the number of CART+/GAL+ nerve fibers in the particular muscle layers, and taking into consideration the proposed neuromodulatory role of both peptides in gastric emptying and acid secretion, as well as neuroprotective role in the ENS. However, further studies are necessary to elucidate, in detail, the exact role of CART and GAL in cancer-affected human stomach.

## Figures and Tables

**Table 1:** The number of protein gene-product 9.5-immunoreactive (PGP 9.5-IR) neurons counted inside studied myenteric plexi (MP) located in the cancer-affected wall and wall distal from cancer invasion. Data were pooled and presented as the representative populations for 10 patients.

The Number of PGP 9.5-IR Neurons Counted Inside Studied MP	
Total number of PGP 9.5-IR neurons	1265 [100%]
Cancer-affected tissue	601 [47.5%]
Surgical margin	664 [52.5%]

**Table 2:** Immunoreagents used in the present study.

Antisera	Code	Host Species	Dilution	Supplier
Primary Antibody				
CART (61–102)	H-003-61	Rabbit	1:6000	Phoenix Pharmaceuticals, Inc., Burlingame, CA, USA
Galanin	T-5034	Guinea pig	1:1200	Bachem AG, Bubendorf, CH
PGP 9.5	7863-2004	Mouse	1:950	Biogenesis, Kingstone, NH, USA
**Secondary Antibody**				
Biotinylated polyclonal anti-rabbit	E0432	Goat	1:1000	Dako, Glostrup, DK,
Fluorescein-conjugated AffiniPure anti-guinea pig	706-096-148	Donkey	1:450	Jackson Immunoresearch, West Grove, PA, USA
AMCA-AffiniPure anti-mouse	715-156-151	Donkey	1:75	Jackson Immunoresearch, West Grove, PA, USA
Cy^TM^3-conjugated streptavidin	016-160-084	-	1:4500	Jackson Immunoresearch, West Grove, PA, USA

## Abbreviations

CART	cocaine- and amphetamine-regulated transcript
GAL	galanin
PGP 9.5	protein gene-product 9.5
IR	immunoreactive
+	positive
-	negative
CRC	colorectal cancer
ENS	enteric nervous system
CNS	central nervous system
MP	myenteric plexi
LMM	lamina muscularis mucosae
CML	circular muscle layer
LML	longitudinal muscle layer
